# Computed tomography‐defined body composition as prognostic markers for unfavourable outcomes and in‐hospital mortality in coronavirus disease 2019

**DOI:** 10.1002/jcsm.12868

**Published:** 2022-01-12

**Authors:** Hans‐Jonas Meyer, Andreas Wienke, Alexey Surov

**Affiliations:** ^1^ Department of Diagnostic and Interventional Radiology University of Leipzig Leipzig Germany; ^2^ Institute of Medical Epidemiology, Biostatistics, and Informatics Martin‐Luther‐University Halle‐Wittenberg Halle (Saale) Germany; ^3^ Department of Radiology and Nuclear Medicine University of Magdeburg Magdeburg Germany

**Keywords:** Meta‐analysis, Systematic review, Sarcopenia, Visceral fat area, COVID‐19

## Abstract

**Background:**

Low skeletal muscle mass (LSMM) and visceral fat areas can be assessed by cross‐sectional images. These parameters are associated with several clinically relevant factors in various disorders with predictive and prognostic implications. Our aim was to establish the effect of computed tomography (CT)‐defined LSMM and fat areas on unfavourable outcomes and in‐hospital mortality in coronavirus disease 2019 (COVID‐19) patients based on a large patient sample.

**Methods:**

MEDLINE library, Cochrane, and Scopus databases were screened for the associations between CT‐defined LSMM as well as fat areas and in‐hospital mortality in COVID‐19 patients up to September 2021. In total, six studies were suitable for the analysis and included into the present analysis.

**Results:**

The included studies comprised 1059 patients, 591 men (55.8%) and 468 women (44.2%), with a mean age of 60.1 years ranging from 48 to 66 years. The pooled prevalence of LSMM was 33.6%. The pooled odds ratio for the effect of LSMM on in‐hospital mortality in univariate analysis was 5.84 [95% confidence interval (CI) 1.07–31.83]. It was 2.73 (95% CI 0.54–13.70) in multivariate analysis. The pooled odds ratio of high visceral fat area on unfavourable outcome in univariate analysis was 2.65 (95% CI 1.57–4.47).

**Conclusions:**

Computed tomography‐defined LSMM and high visceral fat area have a relevant association with in‐hospital mortality in COVID‐19 patients and should be included as relevant prognostic biomarkers into clinical routine.

## Introduction

The prevalent coronavirus disease 2019 (COVID‐19) pandemic has spread throughout the world and is considered a serious threat to global health. The clinical course of COVID‐19 is variable. In fact, most patients experience a mild disease course, but a minority rapidly deteriorate to severe or critical illness with intensive care unit (ICU) admission.^1^, ^2^, ^3^, ^4^, ^5^, ^6^ The case fatality rate during the first peak of the pandemic was over 10% in most European countries.^2^ Clearly, early prediction of an unfavourable course of COVID‐19 can be crucial for optimal treatment care, such as early admission to the ICU, intubation, and treatment escalation.

Already established prognostic factors are age and male sex, which are considered strong independent risk factors for death in COVID‐19 patients. Moreover, a shorter period between symptom onset and emergency room presentation is also unfavourable. Some co‐morbidities, such as dementia, heart failure, and peripheral vascular diseases, are also known risk factors.^1^, ^2^, ^3^, ^4^, ^5^, ^6^

Nowadays, the topic of body composition is of emergent interest throughout medicine. Body composition is a method to define different tissue composition of the human body comprising muscle assessment and different fat area calculation, which can characterize the constitution of patients.^7^, ^8^, ^9^, ^10^, ^11^, ^12^ In clinical practice, computed tomography (CT) is usually used to measure low skeletal muscle mass (LSMM) as a surrogate parameter for sarcopenia.^11^, ^12^ These parameters can be calculated as a by‐product, which as of interest, as in many patients CT scans are performed to search for septic foci or for staging purposes.

Of those parameters, sarcopenia is defined as LSMM and can be caused primarily by ageing or secondarily by diseases, malnutrition, and inactivity.^13^, ^14^ The prevalence of sarcopenia increases with age and is reported to be 5–13% in the general population of the sixth and seventh decades and over 50% for patients above 80 years.^13^ LSMM was recently identified to be a prognostic factor in critically ill patients in the intensive care unit, which highlights the importance of the muscle status in these patients.^15^

One axial CT slide of the L3 intervertebral height is used to the quantify muscle area of paraspinal, abdominal wall, and psoas muscle. Routinely acquired parameters include the calculated skeletal muscle area, which is the total amount of muscle tissue of one slide. A more reliable parameter is the skeletal muscle index (SMI), which is the skeletal muscle area divided by the height squared to address the important factor of body height on muscle tissue. SMI can be considered as more standardized.^11^

Less standardization was reached for fat areas.^12^ In most studies, subcutaneous and visceral fat areas (SAT and VAT) of one slide are quantified. The most accurate CT slide, however, is not clear. Some studies use the slide on the level of the umbilical. Especially, VAT is acknowledged as a prognostic parameter in several tumour entities.^9^, ^10^, ^12^ Visceral obesity as an identified prognostic risk factor defined by high VAT has been acknowledged as a crucial factor.

Different methods have been described in the literature to estimate LSMM and fat areas. Nowadays, a semi‐automatic approach is preferred employing defined Hounsfield unit thresholds to measure the amount of muscle and fat area of the CT slide.^10^, ^11^

Yet despite of the promising nature of preliminary reports of these parameters in COVID‐19 patients, these are predominantly based on retrospective single‐centre studies and reliable data are still missing for this pandemic disease.

The purpose of the present systematic review and meta‐analysis was to calculate the impact of LSMM and fat areas for in‐hospital mortality and unfavourable outcomes in COVID‐19 patients.

## Methods

### Data acquisition

**Figure 1:**
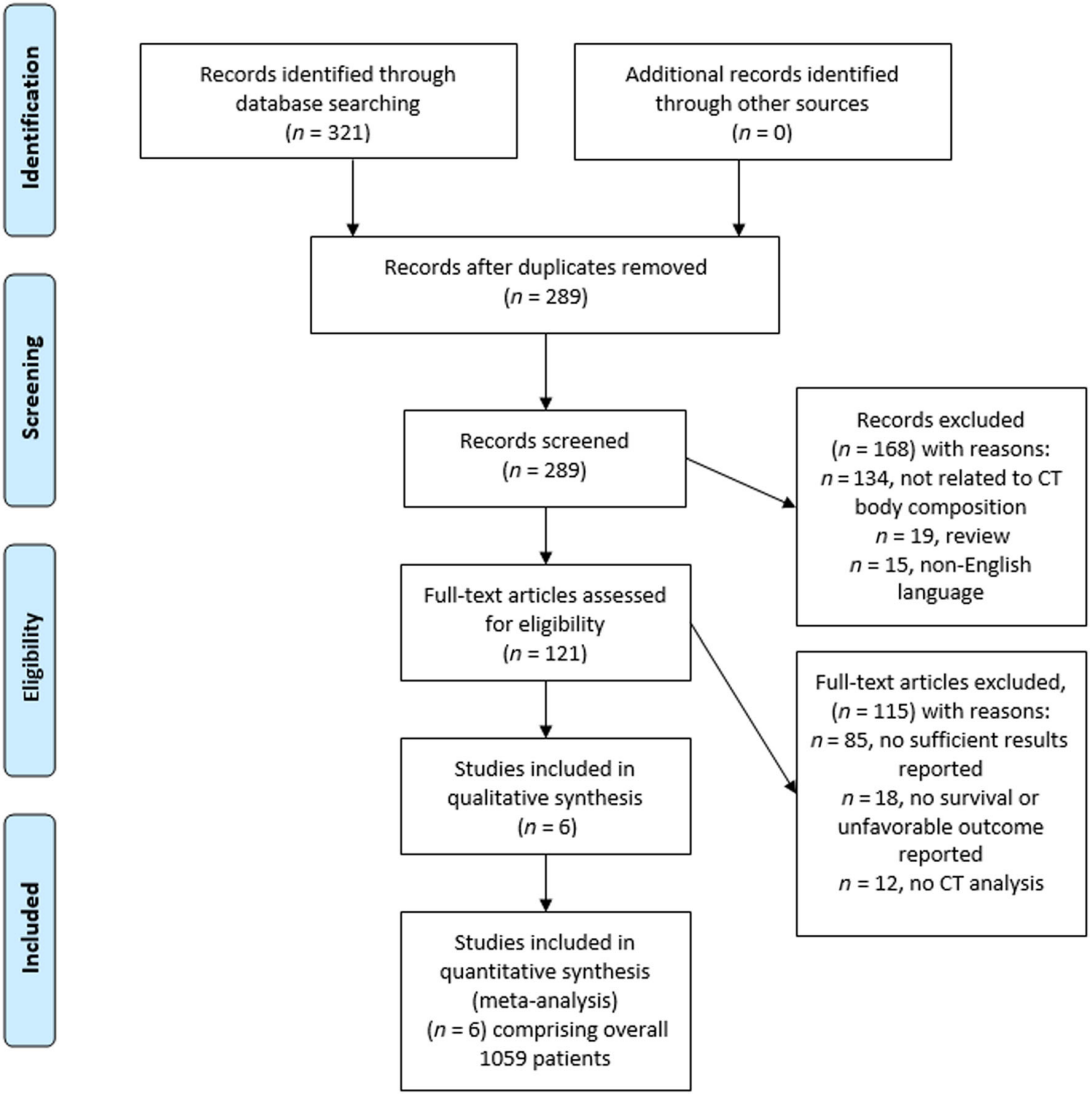
PRISMA flow chart provides an overview of the paper acquisition. Overall, six studies with 1059 COVID‐19 patients were suitable for the analysis.

MEDLINE library, Cochrane, and Scopus databases were screened for LSMM and fat area evaluation in COVID‐19 patients up to September 2021. The paper acquisition is summarized in *Figure* 1.

The following search words were used: ‘COVID‐19’ AND ‘sarcopenia’ OR ‘low skeletal muscle’ OR ‘muscle mass’ OR ‘body composition’ OR ‘fat area’.

The primary endpoint of the systematic review was the odds ratio with reported confidence interval (CI) for LSMM and fat area on unfavourable outcome and in‐hospital mortality. Studies (or subsets of studies) were included, if they satisfied the following criteria: (i) COVID‐19, (ii) LSMM/sarcopenia defined by CT, (iii) visceral and subcutaneous fat area, and (iv) reported odds ratio or hazard ratio with CI. Exclusion criteria were (i) systematic reviews, (ii) case reports, (iii) non‐English language, and (iv) sarcopenia/LSMM/fat areas calculated on other modalities than CT.

The Preferred Reporting Items for Systematic Reviews and Meta‐Analyses (PRISMA) statement was used for the analysis.^16^ In total, six studies were suitable for the analysis and included into the present study.^17^, ^18^, ^19^, ^20^, ^21^, ^22^

### Data extraction

Data extraction was performed by H.‐J. M. followed by an independent evaluation of extractions for correctness (A. S.). For each study, details regarding study design, year of publication, country of origin, patient number, patient characteristics (age and sex), diagnosis, treatment, LSMM definition and prevalence, muscle mass evaluation methods, fat area, threshold values, overall survival outcome results, and adjustment factors were extracted.

### Quality assessment

The quality of the included studies was assessed by the Newcastle–Ottawa Scale.^23^ Study quality assessment was conducted by two authors (H.‐J. M. and A. S.) and mainly included the selection of cases, comparability of the cohort, and outcome assessment of exposure to risks. A score of 0–9 was assigned to each study, and a study with score ≥6 was considered to be of high quality.

### Statistical analysis

The meta‐analysis was performed using RevMan 5.3 (2014; Cochrane Collaboration, Copenhagen, Denmark). Heterogeneity was calculated by means of the inconsistency index *I* ^2^.^24^, ^25^ Finally, DerSimonian and Laird random‐effects models with inverse variance weights were performed without any further correction.^26^ Funnel plot and Egger test were performed for analysis of publication bias.

## Results

### Quality of the included studies

Of the included six studies, all were of retrospective design. *Tables* 1A and 1B give an overview of the included studies.

**Table 1A:** Overview of the included studies investigating LSMM

Authors	Country	Study design	Time period of the study	Mean age (years)	Gender (female), *n* (%)	Included patients, *n*	Patients with LSMM, *n* (%)	Definition of sarcopenia	Calculation of sarcopenia	Defined Hounsfield units for muscle area	Time frame of CT acquisition	Mortality definition
Kim *et al*., 2020	South Korea	Retrospective	17 February to 19 May 2020	62	77 (63.6)	121	29 (24.0)	Below 24 cm^2^/m^2^ for men and 20 cm^2^/m^2^ for women	Every muscle on TH12 level, SMI	0−100	Chest CT at baseline	Hospitality
McGovern *et al*., 2021	UK	Retrospective	17 March to 1 May 2020	67% of patients of over 70 years	33 (52)	63	39 (61.9)	Below 43 cm^2^/m^2^ for men and 41 cm^2^/m^2^ for women when BMI under 25; 53 cm^2^/m^2^ for men and 41 cm^2^/m^2^ for women when BMI over 25	Every muscle on L3 level, SMI	−29 to 150	CT at baseline	30 day mortality
Moctezuma‐Velázquez *et al*., 2021	Mexico	Retrospective	26 February to 15 May 2020	51	187 (36.0)	519	115 (22.0)	Below 42.6 cm^2^/m^2^ for men and 30.6 cm^2^/m^2^ for women when BMI under 25; 53 cm^2^/m^2^ for men and 41 cm^2^/m^2^ for women when BMI over 25	Every muscle on TH12 level, SMI	−29 to 150	Chest CT at baseline	Hospitality
Ufuk *et al*., 2020	Turkey	Retrospective	20 March to 30 April 2020	48	54 (41.5)	130	74 (56.9)	First tertile of PMI values, for men 12.73 cm^2^/m^2^ and for women 9 cm^2^/m^2^	Pectoralis muscle, PMI	−50 to 90	Chest CT at baseline	Hospitality
Yang *et al*., 2021	China	Retrospective	1 January to 30 March 2020	66	73 (51.0)	143	71 (49.7)	Sex‐specified median value as threshold	Every muscle on L3 level, SMI	−29 to 150	Abdominal CT	Critical illness or death

BMI, body mass index; CT, computed tomography; LSMM, low skeletal muscle mass; PMI, pectoralis muscle index; SMI, skeletal muscle index.

**Table 1B:** Overview of the included studies investigating VAT

Authors	Country	Study design	Time period of the study	Mean age (years)	Gender (female), *n* (%)	Included patients, *n*	Patients with high VAT, *n* (%)	Threshold value for high VAT	Calculation of VAT	Defined Hounsfield units for fat area	Time frame of CT acquisition	Outcome
Favre *et al*., 2020	France	Retrospective	Not stated	63.6	44 (40.0)	112	32 (29.0)	128.5 cm^2^	L3 level, VAT	Not stated	Not stated	Severe course
McGovern *et al*., 2021	UK	Retrospective	17 March to 1 May 2020	67% of patients of over 70 years	33 (52)	63	42 (66.7)	>160 cm^2^ for men and 80 cm^2^ for women	L3 level, VAT	−190 to 30	CT at baseline	30 day mortality
Yang *et al*., 2021	China	Retrospective	1 January to 30 March 2020	66	73 (51.0)	143	73 (51.0)	100 cm^2^	L3 level, VAT	−190 to 30	Abdominal CT	Severe course

CT, computed tomography; VAT, visceral fat area.

The overall risk of bias can be considered as low, indicated by the high Newcastle–Ottawa Scale values throughout the studies (*Table* 2). The only concerns for bias were one study,^17^ which did not report sufficiently how the fat areas were measured, and no clear statement, when the patients suffered from COVID‐19.

**Table 2:** The quality of the studies defined by Newcastle–Ottawa Scale

Study	Is the case definition adequate	Representativeness of the cases	Selection of controls	Definition of controls	Comparability of cases and controls on the basis of the design or analysis	Ascertainment of exposure	Same method of ascertainment for cases and controls	Non‐response rate	Quality score
Favre *et al*., 2020			*	*	*	*	*	*	6
Kim *et al*., 2020	*	*	*	*	*	*	*	*	8
McGovern *et al*., 2021	*	*	*	*	*	*	*	*	8
Moctezuma‐Velázquez *et al*., 2021	*	*	*	*	*	*	*	*	8
Ufuk *et al*., 2020		*	*	*	*	*	*	*	7
Yang *et al*., 2021		*	*	*	*	*	*	*	7

The asterisk stands for a positive point of the study per category.

**Figure 2:**
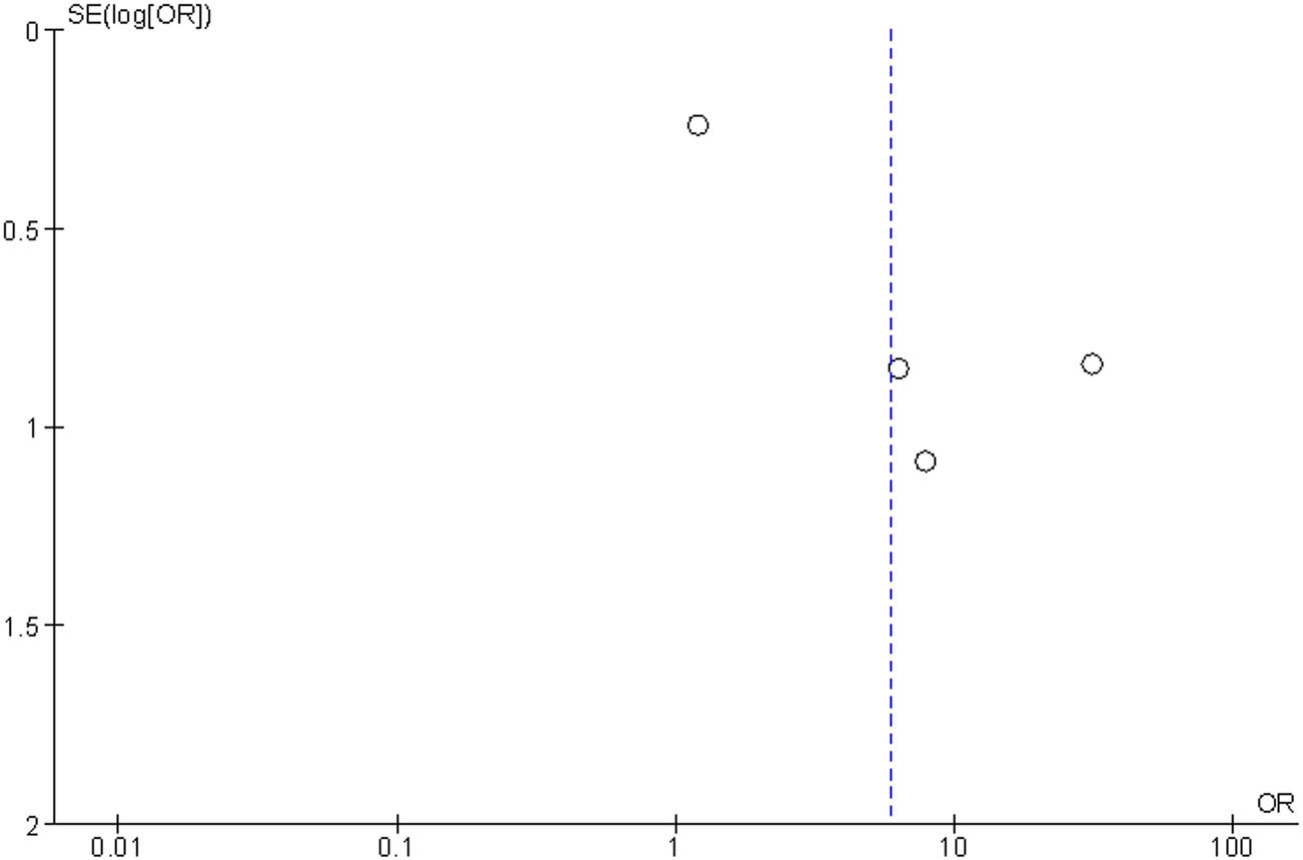
Funnel plot. No statistically significant publication bias can be identified.

Egger test could not identify significant bias (*P* = 0.089). *Figure* 2 displays the corresponding funnel plot.

### Patients

The included studies comprised over all 1059 patients. There were 591 men (55.8%) and 468 women (44.2%), with a mean age of 60.1 years ranging from 48 to 66 years.

In all studies, COVID‐19 was estimated on RT‐PCR. Five studies investigated patients during the first wave of the pandemic, and one study did not report the exact time period.^17^ Three studies (50%) were performed in Asia, two studies (30%) in Europe, and one study (10%) in South America.

### Prevalence of low skeletal muscle mass

A total of 976 patients were analysed in the analysis of LSMM on COVID‐19 patients.

There were 648 patients with no LSMM (66.4%) and 328 patients with LSMM (33.6%).

Different methods were employed for measurement of LSMM (*Table* 1A). The SMI on the level of TH12 was used in two studies (40%)^18^, ^20^; in two studies (40%), the common SMI on the level L3 was used^19^, ^22^; and in one study, the area of the pectoralis muscle was measured (20%).^21^

### Influence of low skeletal muscle mass on clinical outcomes

**Figure 3:**
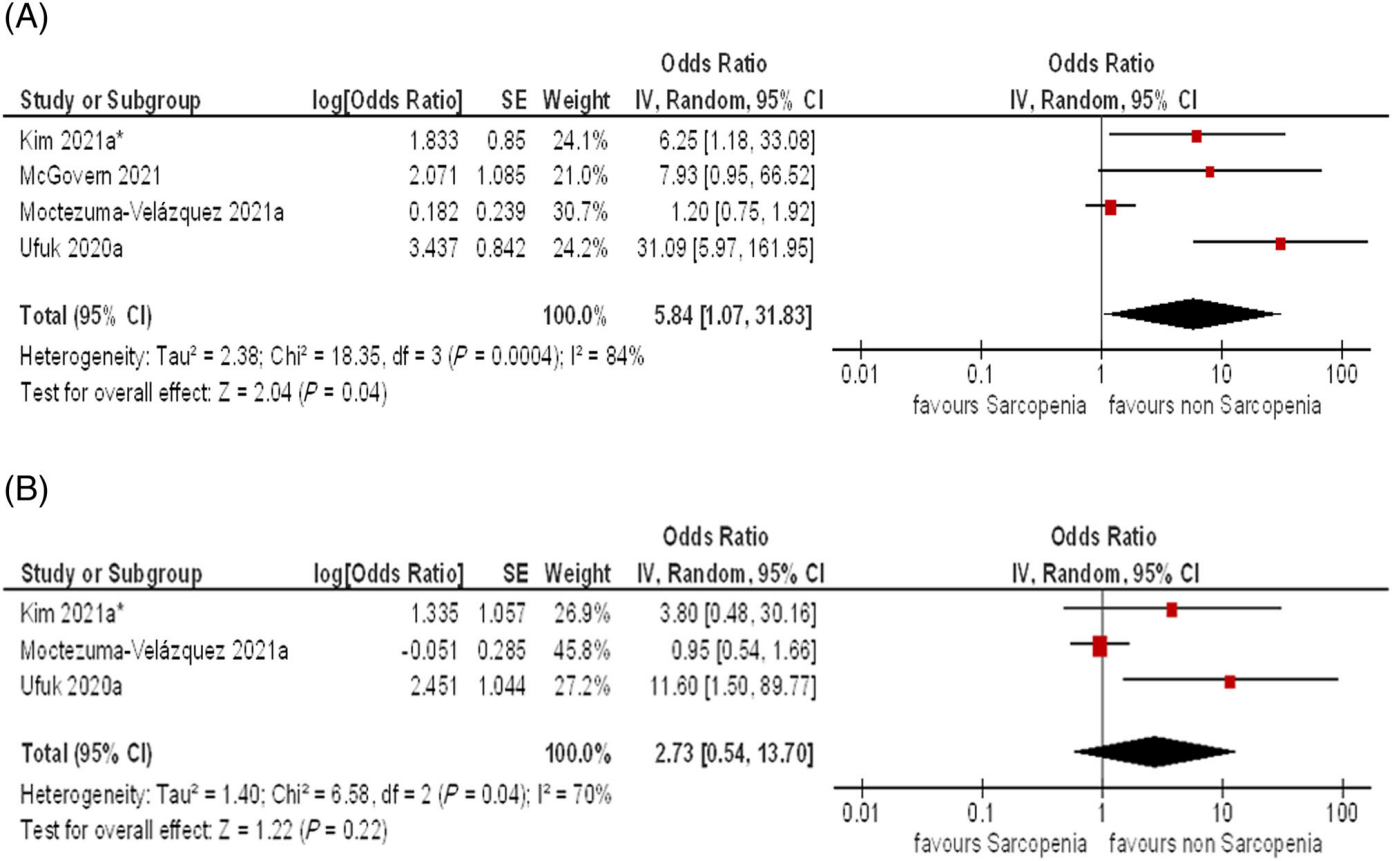
*(A)* Forest plots of the odds ratios for the effect of LSMM on in‐hospital mortality in univariate analysis. The pooled odds ratio was 5.84 (95% CI 1.07–31.83). Kim *et al*. reported hazard ratios. *(B)* The pooled odds ratio for the effect of LSMM on in‐hospital mortality in multivariate analysis was 2.73 (95% CI 0.54–13.70).

Overall, four studies with 976 patients were suitable for the analysis between LSMM and in‐hospital mortality. LSMM was associated with in‐hospital mortality in patients with COVID‐19. The pooled odds ratio for the effect of LSMM on in‐hospital mortality in univariate analysis was 5.84 (95% CI 1.07–31.83, *τ* ^2^ = 2.38, *χ* ^2^ = 18.35, df = 3, *I* ^2^ = 84%) (*Figure* 3A). In multivariate analysis, it was 2.73 (95% CI 0.54–13.70, *τ* ^2^ = 1.40, *χ* ^2^ = 6.58, df = 2, *I* ^2^ = 70%) (*Figure* 3B).

**Figure 4:**
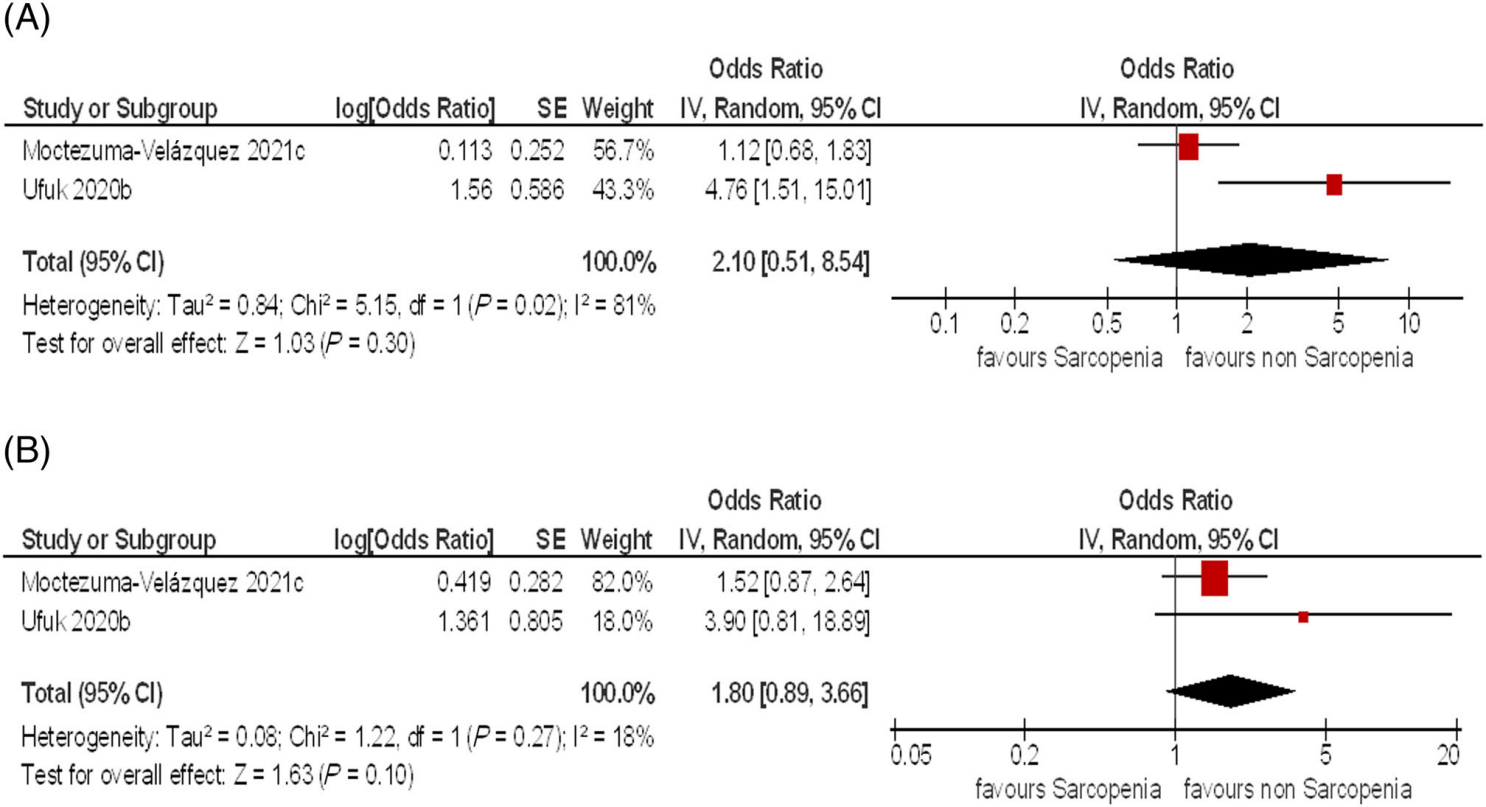
*(A)* Forest plots of the odds ratios for the effect of LSMM on need of mechanical ventilation. The pooled odds ratio for the effect of LSMM on need of mechanical ventilation in univariate analysis was 2.1 (95% CI 0.51–8.54). *(B)* The pooled odds ratio for the effect of LSMM on need of mechanical ventilation in multivariate analysis was 1.8 (95% CI 0.89–3.66).

Associations between LSMM and need for mechanical ventilation were analysed in two studies with 649 patients. The pooled odds ratio in univariate analysis was 2.1 (95% CI 0.51–8.54, *τ* ^2^ = 0.84, *χ* ^2^ = 5.15, df = 1, *I* ^2^ = 81%) (*Figure* 4A). In multivariate analysis, it was 1.8 (95% CI 0.89–3.66, *τ* ^2^ = 0.08, *χ* ^2^ = 1.22, df = 1, *I* ^2^ = 18%) (*Figure* 4B).

**Figure 5:**
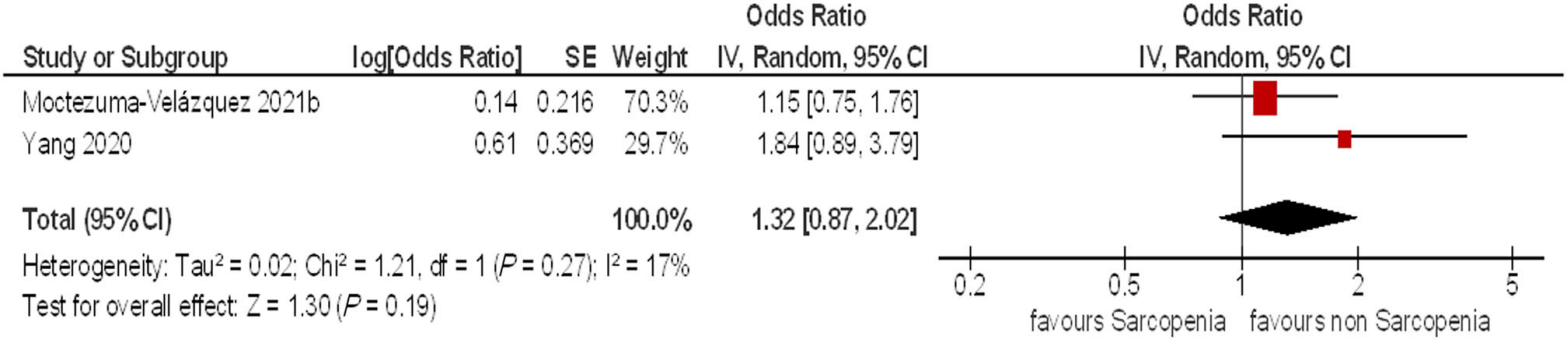
Forest plots of the odds ratios for the effect of LSMM on ICU admission. The pooled odds ratio for the effect of LSMM on ICU admission in univariate analysis was 1.32 (95% CI 0.87–2.02).

Finally, two studies with 662 patients were suitable for the analysis between LSMM and ICU admission. The pooled odds ratio for the effect of LSMM on ICU admission in univariate analysis was 1.32 (95% CI 0.87–2.02, *τ* ^2^ = 0.02, *χ* ^2^ = 1.21, df = 1, *I* ^2^ = 17%) (*Figure* 5).

### Influence of visceral fat area on unfavourable outcome

**Figure 6:**
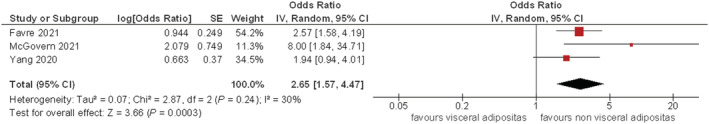
Forest plots of the odds ratios for the effect of high VAT on unfavourable outcome. The pooled odds ratio in univariate analysis was 2.65 (95% CI 1.57–4.47).

Three studies with 288 patients were included into the analysis between VAT and unfavourable outcome.^17^, ^19^, ^22^ Favre *et al*. investigated the outcome of ICU admission and critical illness, McGovern *et al*. investigated the 30 day mortality, and Yang *et al*. investigated the outcome of need of ventilation and death. The pooled odds ratio of high VAT on unfavourable outcome in univariate analysis was 2.65 (95% CI 1.57–4.47, *τ* ^2^ = 0.07, *χ* ^2^ = 2.87, df = 2, *I* ^2^ = 30%) (*Figure* 6).

## Discussion

This is the first meta‐analysis about the influence of LSMM and VAT derived from CT on unfavourable outcomes and in‐hospital mortality in COVID‐19 patients. As shown, there was a significant effect for LSMM and high VAT on mortality and unfavourable outcomes in univariate as well as multivariate analyses. These findings highlight the importance of body composition assessment in patients with COVID‐19 infection.

Coronavirus disease 2019 has a high mortality in patients with an unfavourable course. Thus, a short‐term mortality of up to 20% was reported in COVID‐19 patients of the ICU.^1^, ^2^, ^3^, ^4^, ^5^, ^6^ Already established prognosis parameters are age, male sex, and shorter time period between symptom onset and the admission to the emergency room.^1^, ^2^, ^3^, ^4^, ^5^, ^6^, ^27^, ^28^, ^29^, ^30^, ^31^, ^32^ Moreover, the consolidation extension of CT images is also considered prognostic relevant and can be considered the most important factor derived from radiological images to date.^2^ The present analysis can support the importance of novel body composition CT parameters for prognostic purposes. CT images can provide prognostic biomarkers, which reach beyond the quantification of pulmonary consolidation.

For clinical parameters, several scores were proposed to predict mortality in COVID‐19.^31^, ^32^ A recent study could show that a score based on serologically parameters, white blood cells, C‐reactive protein, lymphocyte ≥0.8 × 10^9^/L, and lactate dehydrogenase ≥400 U/L was highly accurate with an area under the curve of 0.95.^32^ Of great interest could be whether imaging biomarkers could provide complementary information additionally to serologically parameters. There is definite need of further studies to combine both prognostic fields to elucidate this hypothesis.

The topic of body composition is an emergent field of research.^7^, ^8^, ^9^, ^10^, ^13^, ^14^ Of note, there is extensive literature regarding possible applications and interesting prognostic implications of LSMM and fat areas around medicine.^7^, ^8^, ^9^, ^10^, ^11^, ^12^, ^13^, ^14^ One should consider that LSMM and fat area calculations are easily made from every CT image without additional scan time or cost. Almost all patients in critical state are at potential risk of skeletal muscle loss due to prolonged bed rest and systematic inflammation.^13^ Especially elderly patients with primary sarcopenia are more at risk for associated muscle wasting than patients without.^13^

Considerably, there are also great variations between studies in regard of estimation of different body composition parameters. One of the most important parameters of LSMM is the SMI. This index uses the muscle area on the L3 level and the body height to perform a reliable estimation of LSMM.^11^, ^13^ Most commonly, a semi‐automatic measurement was performed utilizing Hounsfield unit thresholds to quantify the muscle and fat areas. Presumably, the semi‐automatic approach might be more reliable and with less inter‐reader variability.

Notably, most studies in the LSMM analysis used surrogate parameters derived from chest CT.^18^, ^20^, ^21^ These were calculated on the TH12 level,^18^, ^20^ which has been shown to be strongly correlated with the muscle area of L3 level.^33^ Therefore, LSMM parameter of TH12 can be a good surrogate parameter for the already established parameter SMI on L3 level. One study, however, utilized the muscle area of the pectoralis muscle, which might be not a surrogate parameter for L3 level and should be considered as a slightly different LSMM parameter.^21^ This can also be accounted for the high heterogeneity identified in the analyses.

Moreover, there might be differences caused by the different patient samples of the studies of different continents. The patient samples might also have slightly different associated risk factors and co‐morbidities, which should be considered with care, when discussing the present results.

Low skeletal muscle mass was associated with mortality as well as prolonged intubation duration, airway complications, and weaning failure in several studies of critically ill patients.^15^, ^34^, ^35^ According to Woo *et al*.,^34^ decreased skeletal muscle mass was associated with extubation failure after long‐term mechanical ventilation for more than a week based upon a study on 45 patients. The authors conclude that it could be important to diagnose decreased skeletal muscle mass in critically ill patients to reduce extubation failure rates.^34^ It appears logically that LSMM has an influence in such an important aspect of critical care, which might also result in the association with mortality.

The investigated unfavourable outcomes like mechanical ventilation and ICU admission have also a relevant impact on mortality with odds ratios of 2.08 and 3.8 in a recent multivariate analysis,^35^ which strengthens the importance of the significant results in the subanalyses of the present analysis.

Regarding VAT, the importance of visceral obesity was clearly shown in several diseases.^36^ Visceral fat is considered harmful because it produces pro‐inflammatory cytokines released directly into the bloodstream and can lead to cytokine production called ‘cytokine storms’. The link between severity of COVID‐19 infection and fat distribution is supported by the angiotensin‐converting enzyme‐2, which is used by SARS‐CoV‐2 virus as a gateway into the body and is overexpressed in visceral fat tissue.^37^ That is why high VAT can be considered as an important prognostic factor in COVID‐19 patients.

The importance of epicardial fat as a special type of fat area, associated with inflammation processes, was also highlighted for COVID‐19 assessment. In a recent investigation, epicardial fat volume was independently associated with mortality and extension of pneumonia.^38^ Unfortunately, we could not perform a subanalysis for epicardial fat, as the data provided by the published studies are too heterogeneous to be pooled in a meta‐analysis. Moreover, we could not include another recent study regarding body composition in COVID‐19, as no dichotomization of the investigated parameters was performed.^39^

The included studies investigated only patients of the first wave of the pandemic, which has a relevant impact on the results. Because of less experience with care of COVID‐19 patients and less knowledge of the disease in general, the course of COVID‐19 patients might be worse than patients of the recent months. This was confirmed in a recent study, which compared mortality of the first and second waves in Barcelona, Spain.^40^ As a key finding, it was shown that first‐wave patients had a more than two‐fold higher mortality compared with second‐wave patients. Moreover, unfavourable outcomes including ICU admission and mechanical ventilation were significantly higher in first‐wave patients compared with the second wave.^40^

One key finding of the present analysis is that there is definite need for new analyses investigating body composition parameter for recent COVID‐19 patients.

The present meta‐analysis has several limitations to address. First, it is composed of published studies with inhomogeneities between studies in regard of measurements and different patient samples. Second, there is the restriction to English language. Third, clinical outcomes were slightly different between studies resulting in possible bias. Fourth, the presented results only rely on patient samples of the first wave of the pandemic. Therefore, the results cannot be considered representative for the current state of the pandemic.

## Conclusions

Computed tomography‐defined low skeletal muscle mass and high VATs have a relevant effect on unfavourable outcome and in‐hospital mortality in COVID‐19. This finding should lead into the inclusion of CT‐defined low skeletal muscle mass and VAT quantification as relevant prognostic biomarkers into the clinical routine.

## Conflict of interest

None declared.

## Funding

None.
